# Application of VEGFA and FGF-9 Enhances Angiogenesis, Osteogenesis and Bone Remodeling in Type 2 Diabetic Long Bone Regeneration

**DOI:** 10.1371/journal.pone.0118823

**Published:** 2015-03-05

**Authors:** Christoph Wallner, Jessica Schira, Johannes Maximilian Wagner, Matthias Schulte, Sebastian Fischer, Tobias Hirsch, Wiltrud Richter, Stephanie Abraham, Ulrich Kneser, Marcus Lehnhardt, Björn Behr

**Affiliations:** 1 Department of Plastic Surgery, BG University Hospital Bergmannsheil, Ruhr University Bochum, Bochum, Germany; 2 Department of Plastic Surgery, BG Trauma Hospital Ludwigshafen, University of Heidelberg, Ludwigshafen, Germany; 3 Research Centre for Experimental Orthopaedics, Heidelberg University Hospital, Heidelberg, Germany; Université de Lyon—Université Jean Monnet, FRANCE

## Abstract

Although bone regeneration is typically a reliable process, type 2 diabetes is associated with impaired or delayed healing processes. In addition, angiogenesis, a crucial step in bone regeneration, is often altered in the diabetic state. In this study, different stages of bone regeneration were characterized in an unicortical bone defect model comparing transgenic type 2 diabetic (*db^-^/db^-^*) and wild type (WT) mice *in vivo*. We investigated angiogenesis, callus formation and bone remodeling at early, intermediate and late time points by means of histomorphometry as well as protein level analyses. In order to enhance bone regeneration, defects were locally treated with recombinant FGF-9 or VEGFA. Histomorphometry of aniline blue stained sections indicated that bone regeneration is significantly decreased in *db^-^/db^-^* as opposed to WT mice at intermediate (5 days post operation) and late stages (7 days post operation) of bone regeneration. Moreover, immunohistochemical analysis revealed significantly decreased levels of RUNX-2, PCNA, Osteocalcin and PECAM-1 in *db^-^/db^-^* defects. In addition, osteoclastogenesis is impaired in *db^-^/db^-^* indicating altered bone remodeling. These results indicate significant impairments in angiogenesis and osteogenesis in type 2 diabetic bones. Importantly, angiogenesis, osteogenesis and bone remodeling could be reconstituted by application of recombinant FGF-9 and, in part, by VEGFA application. In conclusion, our study demonstrates that type 2 diabetes affects angiogenesis, osteogenesis and subsequently bone remodeling, which in turn leads to decreased bone regeneration. These effects could be reversed by local application of FGF-9 and to a lesser degree VEGFA. These data could serve as a basis for future therapeutic applications aiming at improving bone regeneration in the type 2 diabetic patient population.

## Introduction

In physiological conditions, bone regeneration is an efficient process that occurs without scar formation. Nevertheless, due to several medical comorbidities bone regeneration may be reduced or diminished [1]. Thus, it is generally accepted that diabetes impairs and/or delays fracture healing [2]. While type 1 diabetes mellitus (T1DM) is characterized by pancreatic β-cell destruction usually leading to absolute insulin deficiency, type 2 diabetes mellitus (T2DM) is accompanied by hyperglycemia in the context of insulin resistance and relative lack of insulin [3]. Epidemiologically, the vast majority of all diabetic patients (approx. 90%) are suffering from type 2 diabetes mellitus. Both type 1 and type 2 diabetes mellitus patients appear to have an increased fracture risk [4,5]. Clinically, detrimental effects on bone fracture healing have been described in diabetic patients [6,7]. Loder et al. showed that diabetic patients without neuropathy have a 1.6 fold delay in fracture healing [7]. Moreover, in patients with diabetes and neuropathy, a higher incidence for non-union was found [1,8]. Thus, there is a high demand in a growing and aging population for further research to develop novel strategies in treating diabetes associated bony defects and impaired bone regeneration.

So far, insights into mechanisms involved in diabetes-associated impairment of fracture healing were largely generated in T1DM animal models. For instance, expression of runt-related transcription factor-2 (Runx-2), the master gene of osteogenesis, was markedly reduced in an intramembranous bone healing T1DM model. These data indicate an impairment of osteoprogenitor cell recruitment in T1DM. Moreover, a distinct reduction of the proliferative capacity of osteoprogenitor cells has been described in rat T1DM fractures [9–11]. The reason for this is largely unknown. However, it has been hypothesized that diabetes might suppress levels of critical growth factors, relevant for proliferation during the early stages of fracture repair [11,12]. Summarized, a large body of experimental work investigated effects and mechanisms in T1DM models. Surprisingly, markedly less is known for the more prevalent T2DM, which is mainly characterized by insulin resistance but also insulin secretory defects, resulting in variable insulin levels [13]. A hint for decreased bone formation in *db* ^-^/*db* ^-^ mice, resembling T2DM, was found in a bacterial stimulated bone loss model, whereupon osteoclastogenesis was decreased while apoptosis was increased [14]. In another study examining the effects of dental implants in T2DM Goto-Kakizaki rats, there was significant impairment of the bone-implant contact and trabecular bone volume as compared to WT [15]. However, mechanisms leading to impaired bone healing in T2DM were rarely investigated and are less understood.

An imbalance of growth factors is a potential cause for reduced bone regeneration observed in the diabetic state. For instance, in a study examining the effects of growth factors as a treatment option, Kawaguchi and coworkers demonstrated that application of fibroblast growth factor-2 (FGF-2) results in improvements of fracture healing in T1DM rats [16]. Additionally, Gandhi and coworkers showed increased bone healing in T1DM rats by percutaneous delivery of platelet rich plasma [12]. However, in this experimental setting a distinct conclusion about the effect of a single growth factor could not be obtained. Moreover, beneficial effects of PDGF-BB on callus formation were demonstrated in a T1DM rat model [17]. Of note, local application of growth factors to accelerate bone regeneration has already been introduced in the clinical setting [18].

A fundamental initial step for successful bone formation is angiogenesis at the injury site [19–21]. Vascularization not only allows the supply of nutrients and growth factors, but also migration of osteoprogenitors [22] and osteoclasts derived from hematopoietic precursors. Surprisingly, little is known about the effects of T2DM on angiogenesis during bone repair. In a calvarial defect model, angiogenesis was mentioned to be impaired in T1DM mice, indicating a detrimental effect of diabetes on angiogenesis during fracture repair. However, the data were obtained by H&E staining [23] and have to be further validated. Moreover, reduced expression levels of VEGF were also indicative for potential impairments in angiogenesis in T1DM animals [12]. In the broader context of tissue regeneration, it has been shown that angiogenesis is compromised in a limb ischemia model in T2DM *db* ^-^/*db* ^-^ mice [24]. Furthermore, in wound healing, beneficial effects for angiogenesis in diabetic *db* ^-^/*db* ^-^ mice have been shown by local VEGFA treatment [25].

Skeletal vascularization and bone remodeling including osteoblasts differentiation and bone resorption by osteoclasts are key steps in bone healing. VEGFA and FGF-9 have been recently shown as major angiogenic and osteogenic promoter to facilitate bone regeneration. For instance, besides its well-known pro-angiogenic capacity, VEGFA also promotes osteogenesis [26] since VEGFA neutralization by antibodies resulted in impaired bone regeneration capacity in a tibial defect model. FGF-9 has been shown to stimulate proliferation of cell populations consisting of mature osteoblasts as well as calvarial bone cells and promotes osteogenesis by activation of several osteogenesis related genes [27,28]. FGF-9 is capable to promote proliferation of cell populations consisting of more mature osteoblasts in bony defects, competent for osteogenic differentiation [29]. Importantly, FGF-9 is essential for both angiogenesis and osteogenesis [28]. In previous studies, a significant increase in bone regeneration by local application of recombinant VEGFA and FGF-9 could be obtained [28,30]. In the current study, we hypothesized that bone regeneration in T2DM is impaired due to decreased angiogenesis and osteogenesis and aimed to reverse these potential impairments by local application of FGF-9 and VEGFA.

## Material and Methods

### Animal Surgery

All animal experiments were approved by the IACUC Landesuntersuchungsamt Koblenz (#23 177–07/G12–7–046) and LANUV NRW (# 84–02.04.2014.A043). Heterozygous Lepr^db^ (*db* ^*+*^/*db* ^-^) mice were obtained from Jackson Laboratory (# 000697) and kept with unlimited access to water and standard laboratory chow. Heterozygous *db* ^*+*^/*db* ^-^ mice on a C57BL/6J background were mated to obtain WT, *db* ^*+*^/*db* ^-^ and *db* ^-^/*db* ^-^ mice. Genotyping for breeding was performed on genomic DNA by restriction enzyme digest after PCR (forward primer: ATGACCACTACAGATGAACCCAGTCTAC; reverse primer: CATTCAAACCATAGTTTAGGTTTGTC) according to Horvat and Bügner [31]. Female littermates at the age of 16 to 20 weeks were used for all experiments. An established murine tibial defect model was performed as previously described [32]. All surgical procedures were performed under inhalation anesthesia with isoflurane (Abbott GmbH). Briefly, after shaving and disinfecting the left leg, an incision was made on the proximal anterior skin surface over the tibia. After splitting the anterior tibial muscle, the tibia was properly exposed. A 1 mm unicortical defect was created on the anterior tibial surface. Animal groups included no treatment, collagen sponge with 1 μl PBS (control group), collagen sponge with 2 μg recombinant mouse VEGF_164_ (Peprotech) and collagen sponge with 2 μg recombinant mouse FGF-9 (R&D Systems) in 1 μl PBS. We have used two subsets in our experiments. For evaluation of physiological bone healing in diabetic and WT condition, experiments were performed without any vehicle (collagen sponge). The second subset implied insertion of collagen sponges with either PBS as control or recombinant protein into the tibial defects. Before closing the wound with 6–0 Prolene interrupted sutures, the anterior tibial muscle was reset into its anatomical position. Each group consisted of at least 7 animals (power analysis: ε: 1.5, statistical power level: 0.8, probability level: 0.05). Euthanasia was performed according to national and international laws and guidelines. Briefly, cervical dislocation was performed after thorough anesthesia to harvest tissue.

### Tissue preparation and histological procedures

Tibiae were harvested at a given time and fixed in 4% paraformaldehyde (Sigma Aldrich) overnight at 4°C and decalcified in 19% EDTA (Applichem) for five days with daily changes of the solution. Samples were then dehydrated and embedded in paraffin and cut into serial sagittal sections (thickness 6–9 μm).

For immunohistochemical stainings of RUNX-2 (rabbit, polycloncal, SantaCruz Biotechnology, sc-10758, 1:50, Runx-2, AB_2184247) and Osteocalcin, (rabbit, polycloncal, SantaCruz Biotechnology, sc-30045, 1:50, Osteocalcin, AB_653627), sections have been incubated at 58°C for 1h and subsequently rehydrated and incubated with 0,125% Proteinase K for 30 min. After a short washing step with PBS, sections have been permeablized with 0.1% Tween 20 for 4 min and treated with blocking solution for 1h. Incubation with primary antibodies followed overnight in blocking solution at 4°C. After washing with PBS, a rabbit biotinylated secondary antibody followed by the AB reagent and NovaRed (Vector Laboratories) was used for detection. For PCNA detection, PCNA Staining Kit (life technologies) has been performed according to manufactures protocols. Images were taken with an AxioImager M2 Imaging System (Zeiss).

For immunohistochemical stainings of platelet endothelial cell adhesion molecule (PECAM-1), antigen retrieval was performed by incubating rehydrated sections with 0,1% Proteinase K (Roche) in 10 mM Tris (pH 6.8) for 10 min at 37°C. Sections were blocked (10% normal goat serum (NGS, Vector Laboratories)) and permeabilized with 0.1% Triton X-100 (Sigma Aldrich) in PBS for 1h. Next, incubation with primary antibody specific for PECAM-1 (rat, monoclonal, BD Pharmingen, 553370, 1:400, PECAM, AB_394816), was carried out overnight at 4°C. PECAM-1 antibody has been diluted in blocking solution. After washing with PBS, secondary antibody (goat anti-rat conjugated with Alexa 594, Life technologies, 1:1000 dilution in PBS) has been applied and incubated for 4h at room temperature. All sections have been counterstained with DAPI. Sections were subsequently mounted with Fluoromount Aqueous Mounting Medium (Sigma Aldrich). Images for immunofluorescence were taken with a fluorescence microscope (Olympus IX83).

### Quantification of bone formation

Every 6^th^ section was used to characterize bone formation with aniline blue (Roth) staining as previously described [33]. Images were taken with a bright field microscope (Zeiss Axiovert 100, Zeiss) and following settings with Axiovision 4.8: objective 2.5x, exposure time 614 ms, dimensions 3900x3090 Px, scanned color. Histomorphometric measurements of aniline blue stained sections were performed in Adobe Photoshop with modifications [28]. Briefly, a 2000x2000 Px dimensioned selection box was placed to cover the entire defect area. Utilizing the Adobe Magic Wand Tool (settings: tolerance 60%; no-contiguous) new osteoid formation was selected semi-automatically. Tonal separation in two steps and deselecting existing cortical bone resulted in highlighted pixels reliably corresponding to bone formation area. To evaluate indirect and direct ossification Safranin-O staining was performed according to standard procedures [34].

### Assessment of blood vessel sprouting

PECAM-1 positive endothelial cells and blood vessels have been histomorphometrically quantified in a predefined area of 4000x4000 Px. Pixel count was fully automated in Adobe Photoshop by measuring PECAM-1 positive pixels subtracting background staining from controls. This corresponded to the PECAM-1 positive endothelial cells. Blood vessel sprouting was analyzed on at least 4 stained slides per defect by two blinded independent examiners.

### Differentiation and proliferation analysis

Analogous to quantification of bone formation a region of interest was selected (2000x2000 Px). Immunohistochemically RUNX-2 and PCNA positive stained pixels were automatically selected utilizing the Adobe Magic Wand Tool (settings: tolerance 60%; no-contiguous).

### TRAP stainings

Staining of tartrate-resistant acid phosphatase (TRAP) mainly expressed by osteoclasts has been performed according to manufactures protocols (Sigma Aldrich) after deparaffination and rehydration. Sections have been mounted with Aqueous Mounting Medium (Sigma Aldrich). Microscopic analysis was performed with a bright field microscope (Zeiss Axiovert 100, Zeiss) and following settings of the software Axiovision 4.8: objective 2.5x, exposure time 1.2 ms, dimensions 3900x3090 scanned color. For quantification, a 2000x2000 Px dimensioned selection box was placed to cover the defect area in Adobe Photoshop. The Adobe Magic Wand Tool (settings: tolerance 20%; no-contiguous) was used to select all TRAP positive pixels automatically. Additionally, the histogram was used to specifically count red pixels corresponding to TRAP positive pixels.

### Proteome profiler mouse angiogenesis array

WT mice and *db-/db-* mice have been operated as described. 5 mm large areas of the tibia including the defect have been harvested and stored at −80°C until usage. Tissue has been mechanically homogenized and tissue fragments were collected in PBS supplemented with complete protease inhibitor cocktail (Roche). Non-operated WT and *db-/db-* tibiae served as controls. Two tibiae were pooled for analysis. Proteome profiler mouse angiogenesis array (R&D Systems, ARY015) was performed according to manufactures’ instructions. Equal total protein amounts were used for each sample. Protein concentration determination was carried out with BCA protein assay kit (Pierce) according to manufactures´ instructions and ELISA reader ELx800 (Biotek). For quantification, developed x-ray films were scanned, and pixel density of each spot of the array was determined with Image J. For data analysis, average background signal (negative control spots) was subtracted from duplicate spot signal intensity which was subsequently normalized to positive control spots and related to signals from WT mice.

### Statistics

Results of the study are presented as mean ± standard error of the mean (SEM) of at least three independent experiment. P-values were calculated by student’s t-test comparing two groups and ANOVA if comparing more than two groups. Statistical significances were set at a p-value < 0.05.

## Results

### Bone regeneration is decreased in *db* ^-^/*db* ^-^ mice

**Fig 1:**
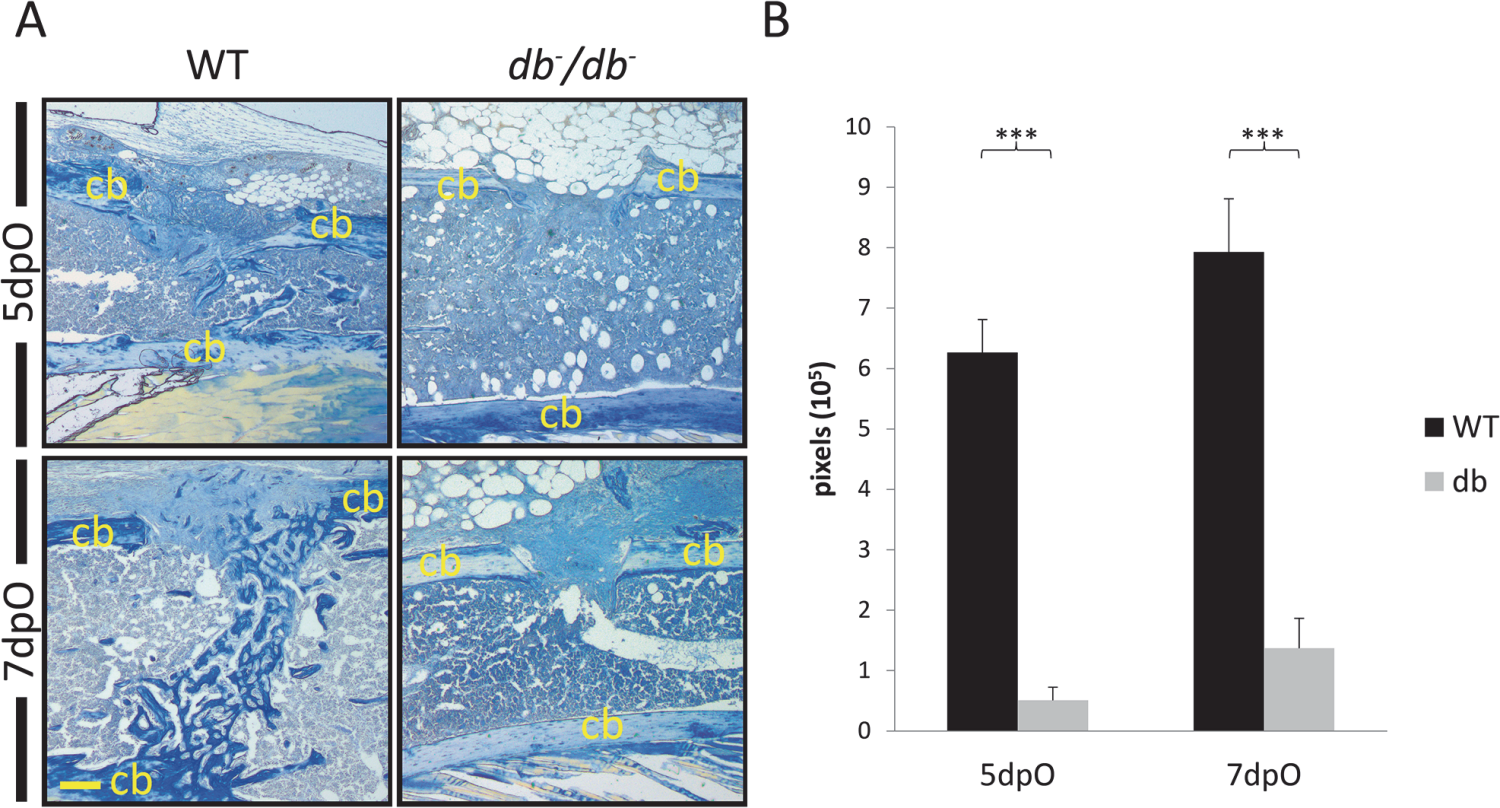
Impaired bone regeneration in a T2DM mice tibia defect model. (A) Aniline blue staining of tibial defects 5 and 7 days post operation (dpO). *db* ^-^/*db* ^-^ mice showed impairments in new bone formation at both time points compared to WT mice. (B) Quantification of aniline blue positive pixels revealed that bone formation is significantly reduced in *db* ^-^/*db* ^-^ mice compared to tibiae of WT mice. Results are shown as ± SEM. P-value: *** < 0.001 (two sample t-test). Scale bar: 200 μm. Cb indicates cortical bone.

At first, we aimed to analyze defined stages of T2DM bone regeneration in an established unicortical tibia defect model. To analyze the regenerative potential, we compared osteoid formation in injured tibiae between WT and *db* ^-^/*db* ^-^ mice 5 and 7 days post operation (dpO) (Fig. 1). Therefore, 1 mm unicortical tibial defects were created and histomorphometry of aniline blue stained sections was performed. 5dpO, bone formation was reduced by 94% in *db* ^-^/*db* ^-^ mice as compared to WT mice. Additionally, bone formation remained significantly decreased 7dpO in *db* ^-^/*db* ^-^ mice by 80% compared to WT mice. Additionally, tibial defects were stained with Safranin-O to exclude endochondral ossification (S1 Fig.). Both groups showed no evidence for endochondral ossification.

### Osteoblast differentiation and proliferation is impaired in *db* ^-^/*db* ^-^ mice

**Fig 2:**
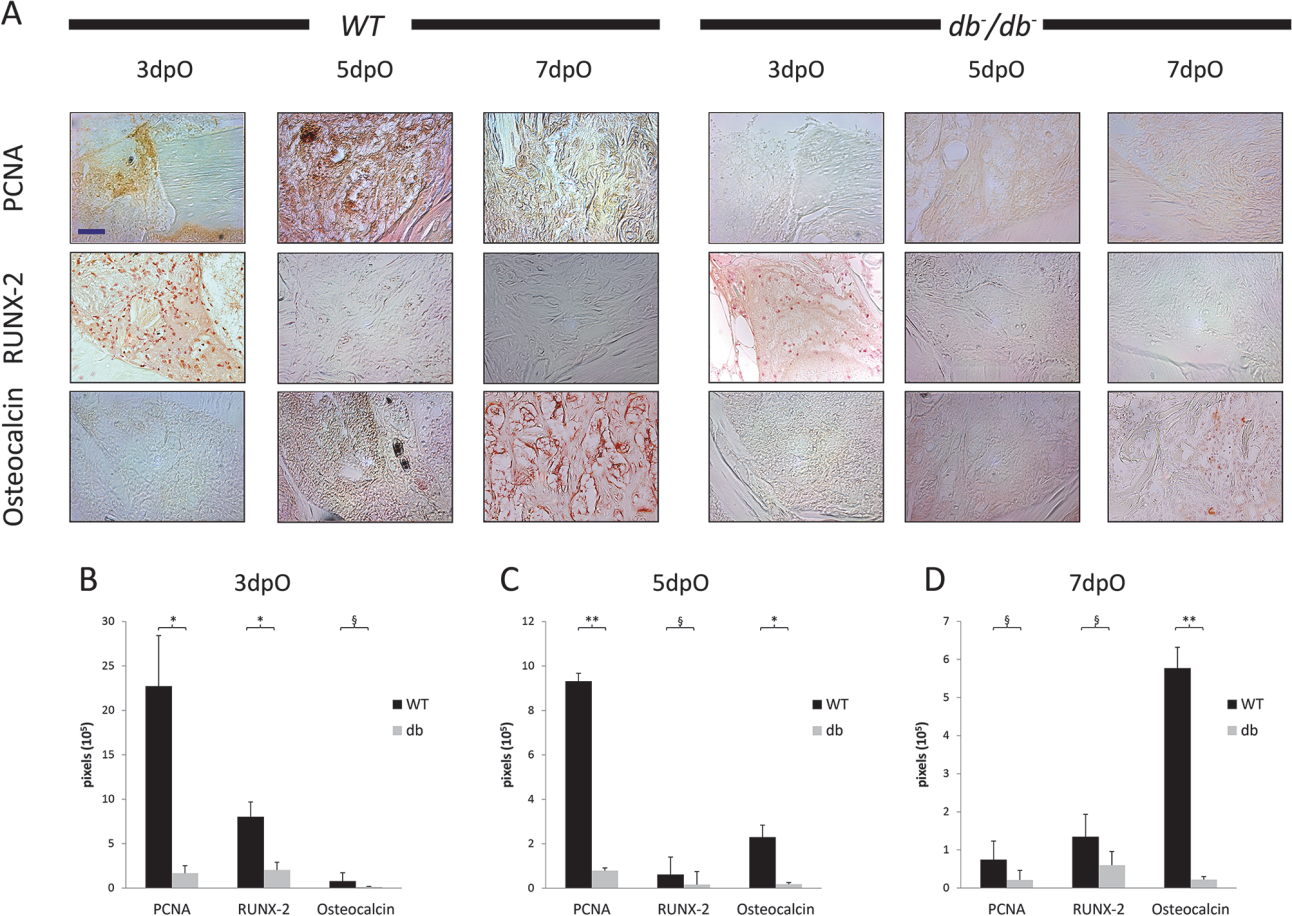
Altered proliferation and differentiation patterns in tibia defects of *db-/db-* mice. (A) Immunohistochemistry for PCNA, RUNX-2, and Osteocalcin was performed at 3, 5 and 7dpO. Compared to WT, *db* ^-^/*db* ^-^ mice showed less osteoblasts and proliferating cells. Moreover, Osteocalcin levels were decreased in *db* ^-^/*db* ^-^ mice at 7dpO. Quantification of Nova Red positive pixel revealed that cell proliferation is significantly reduced in bony defects of *db* ^-^/*db* ^-^ mice compared to tibiae of WT mice in early (B) and intermediate (C) bone regeneration. At early stage, RUNX-2 levels are significantly enhanced in WT mice (B), while Osteocalcin levels are enhanced in intermediate and late bone regeneration (D). Results are shown as ± SEM. P-value: * < 0.05; ** < 0.01; *** < 0.001; § not significant, (two sample t-test). Scale bar: 200 μm.

In order to further characterize impaired bone healing in *db* ^-^/*db* ^-^ mice, immunohistochemical stainings of PCNA, RUNX-2 and Osteocalcin were performed at 3, 5 and 7dpO in *db* ^-^/*db* ^-^ and WT mice. At early and intermediate bone regeneration stages less proliferating PCNA-positive cells could be detected in defects of *db* ^-^/*db* ^-^ compared to WT mice (Fig. 2A). Moreover, decreased RUNX-2 and Osteocalcin levels could be observed 3 and 7dpO, respectively. Quantifications of immunohistochemical stainings confirmed that proliferation is significantly reduced in *db* ^-^/*db* ^-^ mice 3 and 5dpO compared to WT mice (Fig. 2B,C). Moreover, WT mice showed significantly more RUNX-2 positive cells at early stages of bone regeneration (Fig. 2B) as well as significantly higher Osteocalcin levels at intermediate and late stages of bone regeneration compared to *db* ^-^/*db* ^-^ mice (Fig. 2C,D)

### Angiogenesis is impaired in *db* ^-^/*db* ^-^ mice

**Fig 3:**
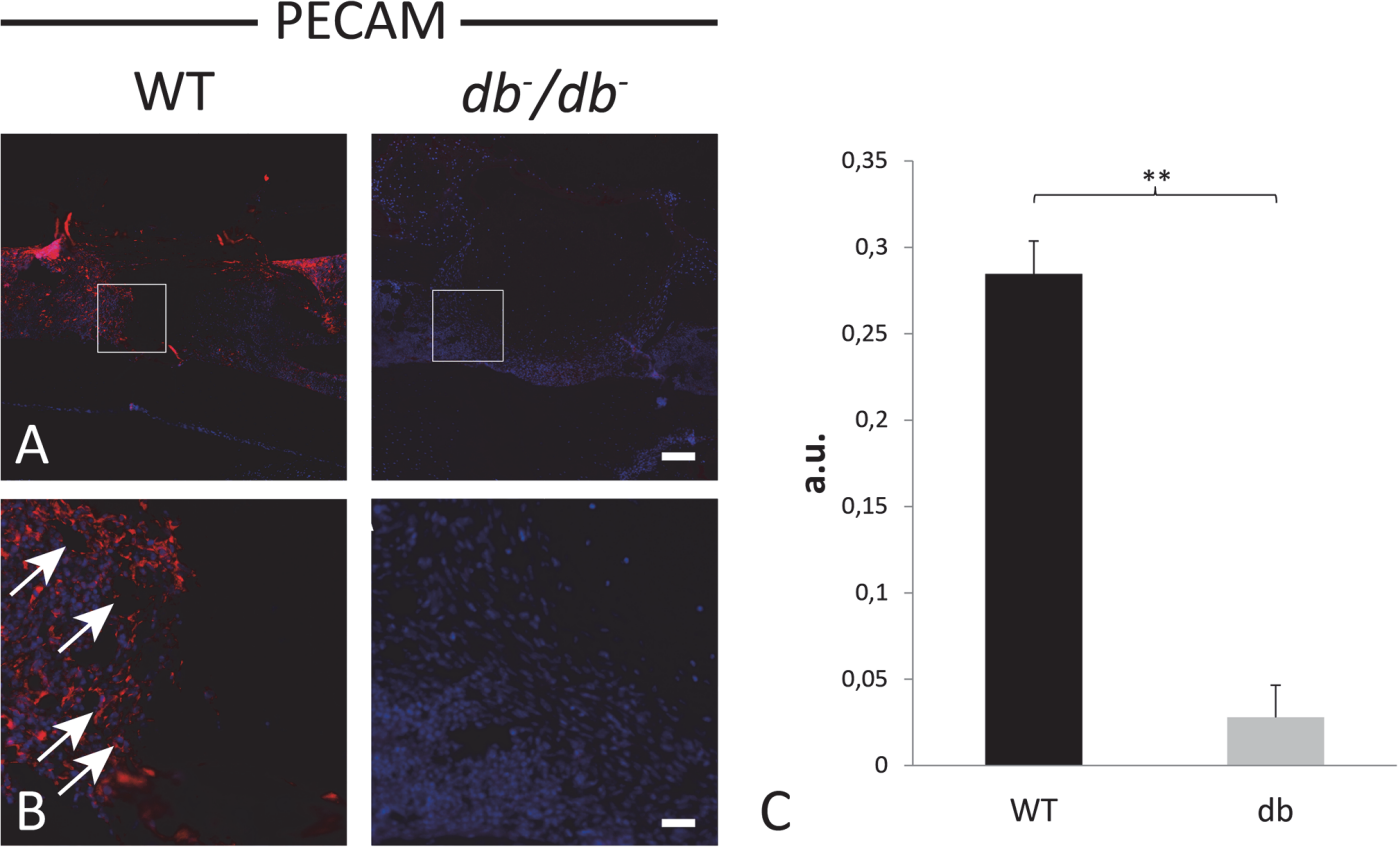
Reduced angiogenesis in *db-/db-* mice tibia defects. (A) Blood vessels and endothelial cells were detected by fluorescence immunohistochemistry for PECAM-1 (3dpO). *db* ^-^/*db* ^-^ mice showed less angiogenesis compared to WT mice. (B) Higher magnifications from (A) (white box). (C) Quantification of PECAM-1 positive pixels (arbitrary units, a.u.) revealed that angiogenesis is significantly reduced in *db* ^-^/*db* ^-^ mice compared to tibiae of WT mice. Results are shown as ± SEM. P-value: ** < 0.01 (two sample t-test). Scale bars: A: 200 μm, B: 45 μm.

It is well known that angiogenesis is a crucial step in early bone healing. In order to investigate the potential impact of T2DM on angiogenesis in bony defects, immunohistochemical stainings for PECAM-1 were performed. We could show that angiogenesis in cortical bony defects is impaired in *db* ^-^/*db* ^-^ mice 3dpO compared to WT mice as indicated by less blood vessels (Fig. 3A,B). Quantification revealed a significant reduction of blood vessel formation by 91% in the defect area of *db* ^-^/*db* ^-^ mice could be confirmed by quantification of PECAM-1 positive pixels (Fig. 3C).

### Decreased osteoclasts invasion into bony defects of *db* ^-^/*db* ^-^ mice

**Fig 4:**
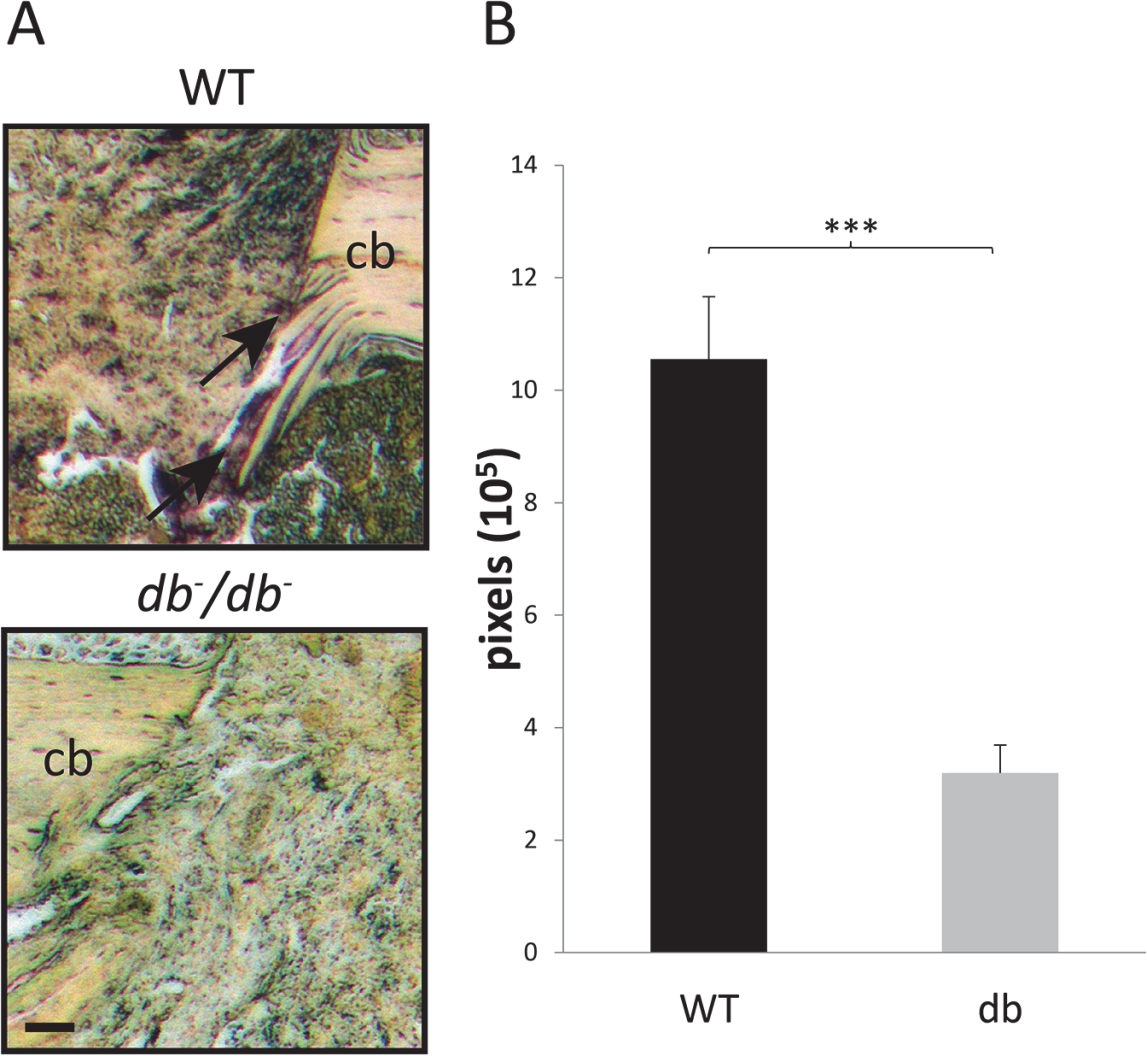
Decreased osteoclasts invasion into bony defects of *db-/db-* mice. (A) TRAP stainings of *db* ^-^/*db* ^-^ and WT mice 7dpO revealed that osteoclasts invasion is decreased in *db* ^-^/*db* ^-^ mice (arrows indicate TRAP-positive pixels) which could be confirmed by quantification of TRAP-positive pixel in the defect area (B). Results are shown as ± SEM. P-value: *** < 0.001 (two sample t-test). Scale bar: 45 μm.

In order to investigate osteoclast invasion in *db* ^-^/*db* ^-^ mice, we performed staining for TRAP, which is highly expressed in osteoclasts. Comparison of TRAP stainings in *db* ^-^/*db* ^-^ and WT mice 7dpO revealed that *db* ^-^/*db* ^-^ mice showed significantly less osteoclast invasion into damaged bone areas (Fig. 4A), which was confirmed by quantification (Fig. 4B).

### FGF-9 application enhances angiogenesis in *db* ^-^/*db* ^-^ mice

**Fig 5:**
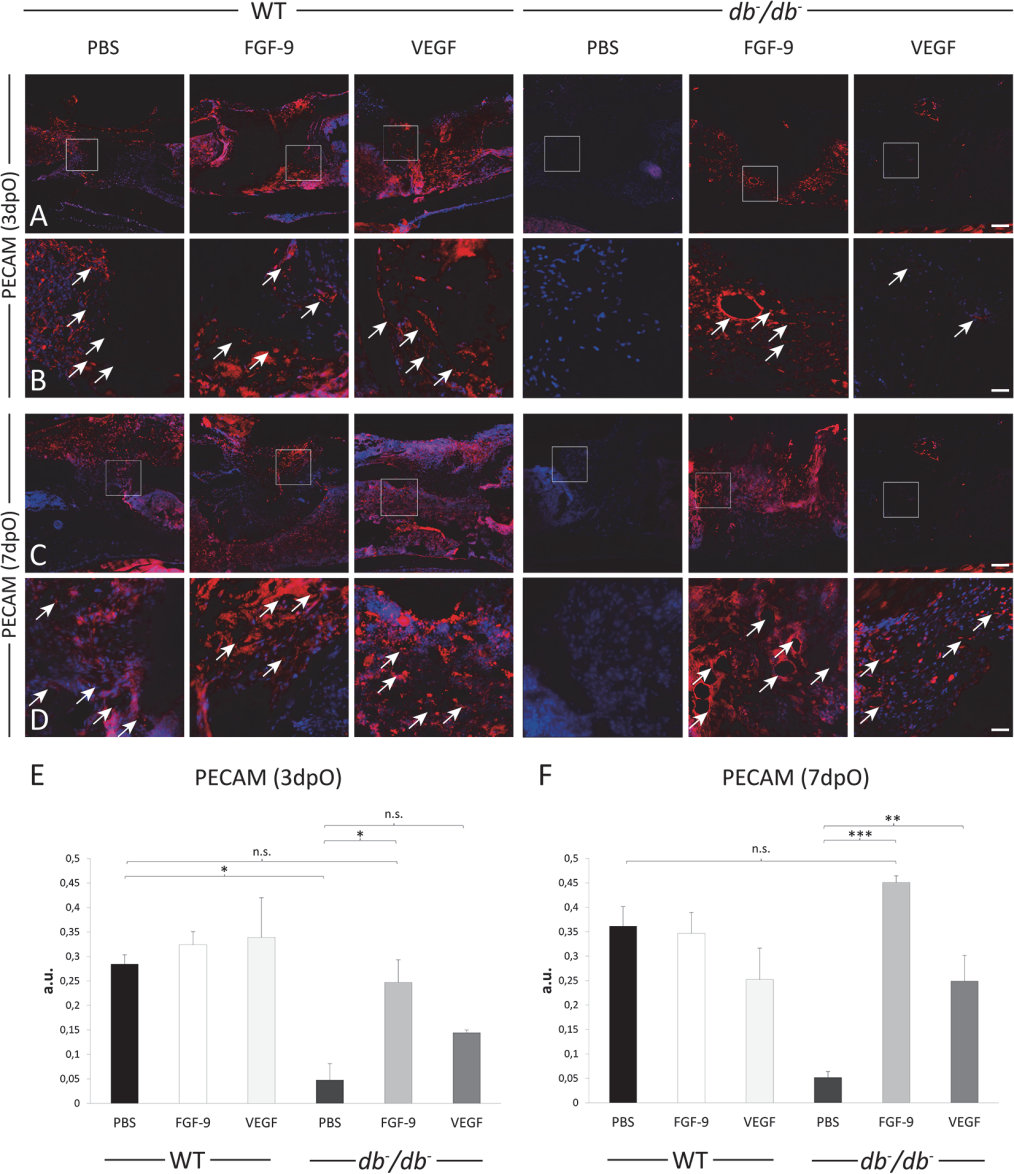
Angiogenesis is enhanced after FGF-9 treatment in *db-/db-* mice. Blood vessels were detected by immunohistochemical staining for PECAM-1 at early (A,B) and late stages (C,D) of bone regeneration. FGF-9 (2 μg) and VEGF (2 μg) treated *db* ^-^/*db* ^-^ mice show enhanced angiogenesis compared to control *db* ^-^/*db* ^-^ mice (PBS). (B and D) higher magnifications of boxed areas from (A) and (C), respectively. Arrowheads indicate PECAM-1 enriched areas identified as vessels. (E) Quantification of PECAM-1 positive pixels revealed that angiogenesis is significantly increased in FGF-9 treated *db* ^-^/*db* ^-^ mice compared to tibiae of control mice whereas VEGF application showed no significant (n.s.) increase in vascular formation (PBS) in early bone regeneration (3dpO). (F) Quantification of PECAM-1 positive pixels in late bone regeneration (7dpO) revealed highly significant enhanced angiogenesis in treated *db* ^-^/*db* ^-^ mice. WT animals treated with recombinant proteins showed no enhanced vascular formation compared to control in early (3dpO) and late bone regeneration (7dpO). Results are shown as ± SEM. P-value* < 0.05; ** < 0.01; *** < 0.001 (two sample t-test). Scale bars: A,C 200 μm; B,D 45 μm.

Our data indicated that both angiogenesis and osteogenesis were markedly reduced in *db* ^-^/*db* ^-^ mice. Given their pro-angiogenic and pro-osteogenic properties, we applied FGF-9 and VEGFA proteins, or PBS (control) soaked collagen sponges to tibial defects in order to reconstitute bone regeneration. We analyzed the effect of VEGFA and FGF-9 application on angiogenesis in bony defects of *db* ^-^/*db* ^-^ and WT mice at early (Fig. 5A,B) and late stages (Fig. 5C,D) of bone regeneration. We found significantly enhanced angiogenesis in *db* ^-^/*db* ^-^ defects treated with FGF-9 at both analyzed time points reaching expression levels equivalent to WT mice. Moreover, VEGFA application led to significantly enhanced angiogenesis at late bone regeneration (7dpO) in *db* ^-^/*db* ^-^ mice.

### Application of FGF-9 and VEGFA enhances osteogenesis and bone remodeling in T2DM bony defects

**Fig 6:**
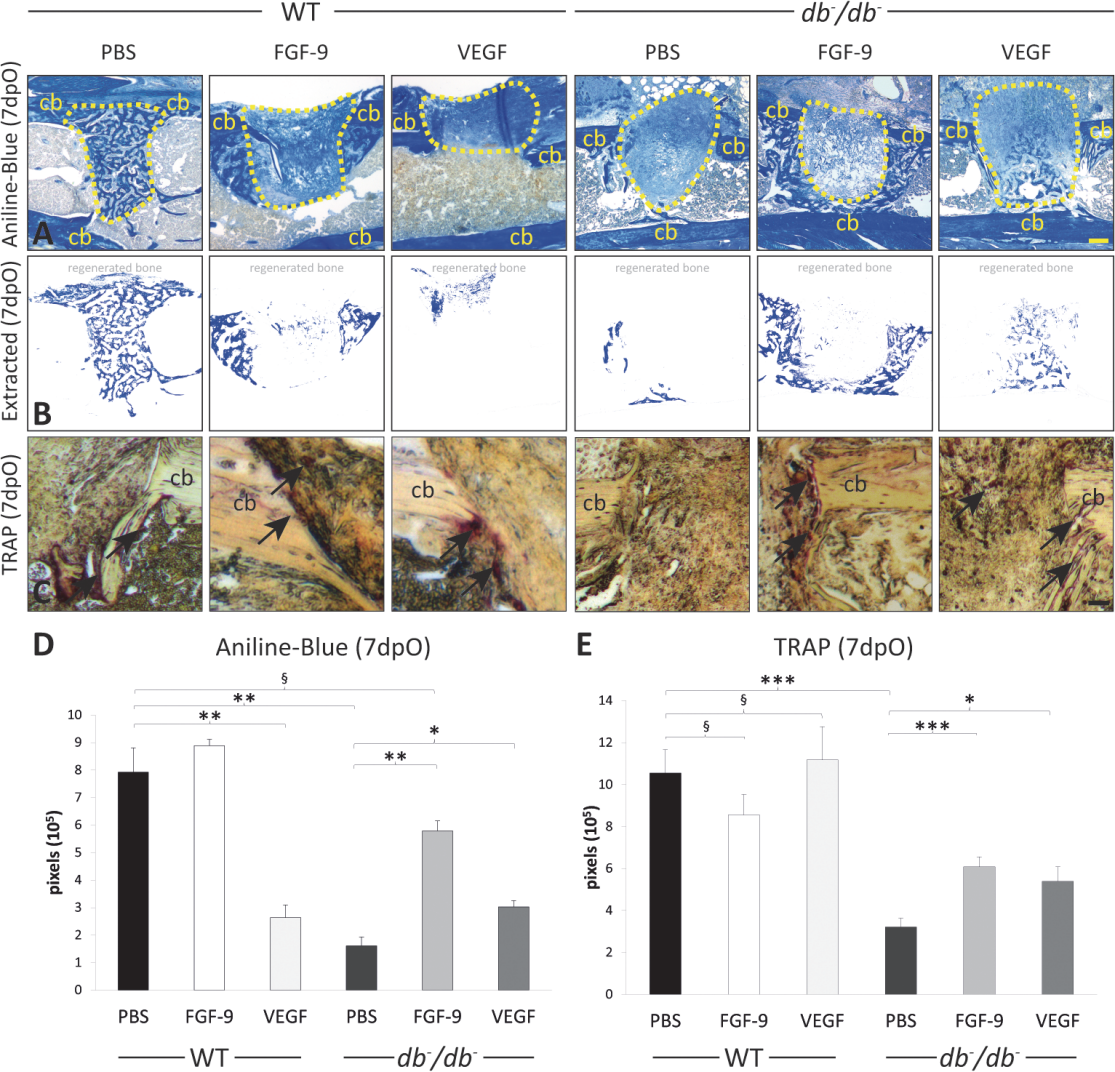
Application of recombinant FGF-9 and VEGFA enhances bone formation and bone resorption in *db-/db-* mice. (A) Aniline blue staining of tibial defects 7dpO of control treated (PBS), FGF-9 and VEGFA treated tibiae of WT and *db* ^-^/*db* ^-^ mice. Application of FGF-9 (2 μg) and VEGFA (2 μg) lead to more osteoid formation compared to the control group (PBS) in *db* ^-^/*db* ^-^ mice. (B) Extracted osteoid formation area. (C) TRAP staining of FGF-9 and VEGFA treated WT and *db* ^-^/*db* ^-^ mice 7dpO revealed that osteoclast invasion into the defect area is enhanced. (D) Quantification of aniline blue positive pixels revealed that bone formation is significantly enhanced by locally applied FGF-9 and VEGF in *db* ^-^/*db* ^-^ mice compared PBS treated diabetic mice. We observed a significant reduction of osteoid formation in VEGF treated WT mice while FGF-9 had no effect on osteoid formation in WT mice. There was no significant difference in osteoid formation of *db* ^-^/*db* ^-^ mice treated with FGF-9 compared to WT mice. (E) Quantification of TRAP-positive pixel resulted in significantly enhanced osteoclast invasion after treatment compared to untreated *db* ^-^/*db* ^-^ *mice*. Results are shown as ± SEM. P-value: * < 0.05; ** < 0.01; *** < 0.001; § not significant (two sample t-test). Scale bars: A: 200 μm, C: 45 μm. Cb indicates cortical bone, yellow dashed line indicates collagen sponge (not traceable in WT PBS).

After having observed an increase in angiogenesis, we next assessed bone regeneration and remodeling. To quantify bone regeneration, we harvested tissue at postoperative day 7 and performed histomorphometry of aniline blue stained sections as indicated. Upon local application of recombinant VEGFA, bone formation was significantly increased in *db* ^-^/*db* ^-^ mice 7dpO (Fig. 6 A,B,D). Importantly, bone regeneration could be further increased by local application of FGF-9 to levels comparable to WT mice. While local application of FGF-9 into WT defects had no effect on bone formation, VEGFA reduced bone formation. Moreover, application of either VEGFA or FGF-9 further enhanced osteoclasts invasion into defects of *db* ^-^/*db* ^-^ mice as indicated by TRAP staining 7dpO (Fig. 6 C,E) which was markedly reduced in *db* ^-^/*db* ^-^ control mice. Of note, application of growth factors had no effect on ostoclastogenesis in WT mice.

### Protein array analysis of *db* ^-^/*db* ^-^ and WT mice

**Fig 7:**
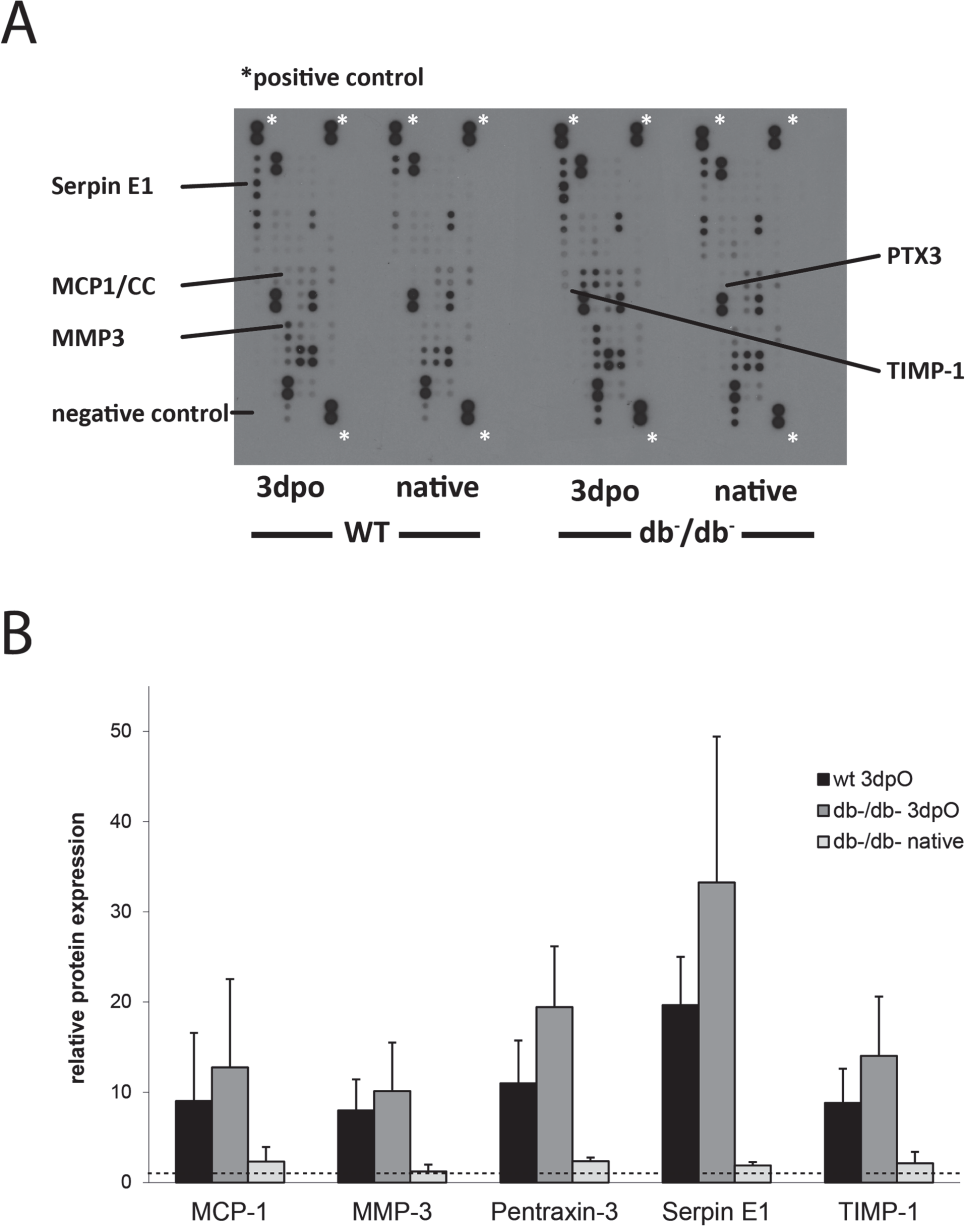
Array analysis of *db* ^-^/*db* ^-^ and WT mice 3dpO. (A) Protein lysates derived from WT and *db* ^-^/*db* ^-^ mice 3dpO and from non-operated native tibiae were run on each array experiment in parallel. (B) Quantification of relative protein levels. Pixel density was normalized to spots of native WT mice after subtraction of background signal. Mean values ± SEM from three independent experiments are given for MCP-1, MMP-3, Pentraxin-3, Serpin E1 and TIMP-1 which are upregulated in operated *db* ^-^/*db* ^-^ mice. Dashed line serve as reference for relative protein levels. Protein levels of WT and *db* ^-^/*db* ^-^ mice 3dpO were not significantly different (two sample t-test).

The experiments performed led to the conclusion that impaired angiogenesis played an important role for the decreased bone regeneration as seen in *db* ^-^/*db* ^-^ mice. In order to investigate angiogenesis upon bone regeneration in further detail, we performed protein array analyses of *db* ^-^/*db* ^-^ and WT mice 3dpO in comparison to uninjured control tibiae from *db* ^-^/*db* ^-^ and WT mice. Equal protein amounts have been used for each sample. Signal intensity of spots were normalized to positive controls and related to signals from WT mice. Data were not statistically significant due to inter-experimental variability, however they do show trends. For instance, our experiments revealed that Pentraxin-3 (PTX3) which is a well-known inhibitor of angiogenesis is upregulated in *db* ^-^/*db* ^-^ mice 3dpO compared to native and operated WT mice (Fig. 7). Moreover, Serpin E1 (Plasminogen activator inhibitor-1, PAI-1) level has been found to be higher in *db* ^-^/*db* ^-^ mice 3dpO as well as MCP-1 (monocyte chemotactic protein-1), MMP-3 (matrix metallopeptidase-3) and TIMP-1 (metallopeptidase inhibitor-1). These proteins are related to angiogenesis but could also play essential roles in osteogenesis and bone remodeling. Angiopoietin-1, CD26, Endoglin (CD105), Endostatin, FGF acidic (FGF-1), hepatocyte growth factor, IGFBP-1, IGFBP-3, MMP-8, MMP-9, IGFBP-9, Osteopontin, CXCL4, SDF-1, Serpin F1 and Thrombonspondin-2 have been found to be expressed in all tissues but without obvious differences (data not shown). All other spots on the array had only background signals.

## Discussion

In the present study, we investigated different stages of bone regeneration in an unicortical tibia defect model comparing *db* ^-^/*db* ^-^ and WT mice. Our experiments revealed that bone regeneration is impaired in *db* ^-^/*db* ^-^ mice due to compromised angiogenesis and osteogenesis. This was accompanied by reduced cell proliferation. Moreover, we showed that osteogenesis-related proteins RUNX-*2* and Osteocalcin are downregulated in *db* ^-^/*db* ^-^ mice and that osteoid formation is impaired in T2DM tibial bone defects. In addition, impaired bone regeneration was accompanied by decreased angiogenesis which could in turn lead to a decrease in osteogenesis. Likewise, osteoclastogenesis seems to be impaired in *db* ^-^/*db* ^-^ mice indicating an imbalanced bone remodeling. Altered protein levels of angiogenesis and bone remodeling related proteins in *db* ^-^/*db* ^-^ mice was further suggested by protein array analysis. Importantly, these pathological conditions could be reversed. As such, FGF-9 application led to an almost complete rescue of bone repair accompanied by significantly increased angiogenesis and bone remodeling.

Insight in cell proliferation is essential for understanding of fracture repair [35]. PCNA, which is synthesized in the late G1 and S phase of DNA replication, is downregulated in the callus in *db* ^-^/*db* ^-^ mice during fracture healing at early and intermediate stages of bone healing compared to WT mice. There are several studies revealing that combination of PCNA and RUNX-2 is indicative for osteoprogenitor cells [27,36,37]. Less proliferating cells indicate impaired bone tissue regeneration in *db* ^-^/*db* ^-^ mice, which could be confirmed by RUNX-2 immunohistochemical stainings 3dpO as well as aniline blue histomorphometry 7dpO.

Most studies investigating bone regeneration in diabetes were previously performed in T1DM models. Although human diabetes is rarely caused by single trigger, monogenic models are commonly used to investigate T2DM. For the purpose of this study, we utilized the well-established leptin receptor knock-out model, mimicking human T2DM conditions including features of the metabolic syndrome [38,39]. In accordance to our data in T2DM, recent studies in animal models of T1DM have demonstrated reduced proliferation in the callus and impaired fracture healing [9]. Moreover, RUNX-2 expression was downregulated followed by decreased Osteocalcin levels in T1DM models [40]. In uninjured *db* ^-^/*db* ^-^ mice, tibiae parameters such as bone volume, bone density, trabecular volume and number of trabeculae were found to be significantly decreased. At the same time, baseline Osteocalcin levels were reduced by 40% in *db* ^-^/*db* ^-^ mice compared to WT mice [41]. The present study revealed that Osteocalcin levels were significantly reduced during bone healing confirming impaired bone regeneration potential in T2DM. In this respect, one could speculate that reduced levels of Osteocalcin in *db* ^-^/*db* ^-^ mice at 5dpO and 7dpO are a consequence of less osteoprogenitor cells present at first. The causes of impaired bone regeneration in T2DM are not fully understood yet. Indeed, a puzzling situation occurs when considering the pro-osteogenic hyperinsulinemia and anti-osteogenic hyperglycemia, which ultimately result in a net detrimental osteogenic effect in T2DM. Insulin is a positive regulator of Osteocalcin activity in osteoblasts and impacts glucose homeostasis by promoting the ability of osteoclasts to enhance bone resorption. Thus, insulin is discussed as a major contributor to bone healing [42–44]. In contrast, hyperglycemia could lead to increased alkaline phosphatase expression and suppressed Osteocalcin, MMP-13, VEGF, and GAPDH levels in osteoblasts resulting in osteoporosis [45]. In the light of our results, Osteocalcin downregulation could result from decreased levels of RUNX-2, which directly regulates Osteocalcin expression as well as from hyperglycemia in *db* ^-^/*db* ^-^ mice. Additionally, hyperglycemia diverts osteoblastic precursor cells to an adipogenic pathway [46]. These physiological alterations in osteoblasts caused by hyperglycemia are mainly regulated by osmotic and non-osmotic pathways [45]. Concluding from the findings of our experiments and the literature, hyperglycemic effects prevail in both, the clinical setting as well as in our diabetic mouse model. Besides dysregulation of RUNX-2, PCNA and Osteocalcin in bony defects of *db* ^-^/*db* ^-^ mice, Serpin E1 was slightly increased in *db* ^-^/*db* ^-^ mice 3dpO as revealed by protein array. Serpin E1 was shown to be involved in the pathogenesis of T1DM osteoporosis in female mice. Elevated Serpin E1 levels could be associated with impaired osteoblast functions in female mice and direct mesenchymal progenitor cells towards adipogenic differentiation [47]. Due to inter-experimental variability, differences were not significant but these experiments indicate that regulation of Serpin E1 deserves further investigations in the context of bone healing in T2DM. Beside reduced osteogenesis, TRAP stainings revealed that osteoclastogenesis is likewise limited in *db* ^-^/*db* ^-^ mice. Osteoclasts differentiate from hematopoietic precursors and invade damaged bone tissue. Given that angiogenesis is reduced in *db* ^-^/*db* ^-^ mice, less precursor cells could invade the defect area resulting in less TRAP positive osteoclasts. Osteoclastogenesis in T2DM is a controversial subject. It has been shown that hyperglycemia lead to aberrant osteoclast differentiation and increased bone resorption [48]. In contrast, in a bacterial stimulated bone loss model in *db* ^-^/*db* ^-^ mice, osteoclastogenesis was found to be decreased [14]. Moreover, *in vitro* experiments indicated that bone fragility typical for diabetes mellitus is not a consequence of excessive bone resorption [49]. It was shown that osteoclasts have a decreased resorption activity under hyperglycemic conditions [50]. Our findings resemble these results suggesting that both, osteogenesis as well as osteoclastogenesis is impaired in *db* ^-^/*db* ^-^ mice.

T2DM and T1DM influence angiogenesis in multiple organs. Reduced angiogenesis could result from impaired vascular endothelial function as shown in patients with diabetes. In bone, regeneration is linked to a correlation and crosstalk between osteoblasts, osteoclasts and endothelial cells. Our experiments revealed a downregulation of the endothelial cell marker PECAM-1 indicating impaired angiogenesis in T2DM bone fractures. Reduction of endothelial progenitor cells (EPCs) is one potential cause for vascular dysfunctions in both T1DM and T2DM [51,52]. Moreover, hyperglycemia has been shown to inhibit microvascular endothelial cell migration during angiogenesis by suppression of RUNX-2 activity in a wound healing model [53]. Protein array further revealed that PTX3 which is a well-known inhibitor of angiogenesis is slightly upregulated in *db* ^-^/*db* ^-^ mice 3dpO. Moreover, MMP-3 and its inhibitor TIMP-1 are upregulated in *db* ^-^/*db* ^-^ mice 3dpO, although the differences seen were not statistically significant. Both molecules are physiologically associated with angiogenesis. In human patients suffering from rheumathoid arthritis, MMP-3 expression is elevated which is directly involved in matrix degradation [54,55]. Moreover, there is a general agreement that diabetes affects MMP regulation in the vasculature [56] and the balance between MMPs and TIMPs [57]. This imbalance between both proteins could lead to dysregulation of angiogenesis as well as bone remodeling in *db* ^-^/*db* ^-^ mice.

Diabetes in general induces reactive oxygen species as well as proinflammatory chemokines and cytokines. We demonstrated that MCP-1 is upregulated in bony defects of db^-^/*db* ^-^ mice. MCP-1 was shown to be highly expressed in adipocytes which are localized in large numbers in the bone marrow of *db* ^-^/*db* ^-^ mice as well as in endothelial cells [58]. MCP-1 leads to oxidative stress, which in turn increases inflammation and endothelial cell apoptosis. Upregulation of MCP-1 could also lead to enhanced expression of TIMP-1 which further results in imbalance of angiogenesis [59].

In our model, local application of VEGF_164_ led to a partial rescue of bone remodeling and vascular formation at late stage and angiogenesis at early stage in *db* ^-^/*db* ^-^ mice. VEGF has been shown to couple hypertrophic cartilage remodeling, ossification and angiogenesis during endochondral bone formation [19]. During bone repair, VEGF is expressed in the fracture callus [60] partially resembling bone development. In a murine model, neutralization of VEGF leads to disruption of femoral fracture regeneration [26]. Importantly, VEGF_164_ seems to play an essential role in bone repair and development as mice exclusively expressing VEGF_164_ develop a normal skeletal phenotype compared to mice expressing other VEGF isoforms than VEGF_164_ [61,62]. Interestingly, application of VEGF into WT bony defects resulted in significantly reduced bone formation. Several studies showed that VEGF promotes ossification by either inducing neovascularisation or by directly affecting bone cells [63]. The effects seen in our experimental setup could be due to an improper dose or simply the fact that bone regeneration in WT mice is already optimal and alterations of the complex orchestra of growth factors may have detrimental effects on healing, however further investigations are required to describe this mechanism.

In previous studies, FGF-9 was shown to be essential in bone development and regeneration [28,64]. Absence of FGF-9 leads to decreased chondrocyte proliferation, delayed initiation of chondrocyte hypertrophy and abnormal osteogenesis secondary to defects in skeletal vascularization [64,65]. In the developing bone, missense mutations in the *Fgf-9* gene lead to joint synostosis and craniosynostosis [66]. Importantly, FGF-9 has also been linked to angiogenesis in bone regeneration. Behr *et al*. identified FGF-9 as an essential protein for angiogenesis and osteogenesis in long bone repair [28]. Moreover, FGF/FGFR signaling has been shown to enhance the intrinsic osteogenic potential by selectively expanding osteogenic cell populations [29]. In our experiments, application of FGF-9 into T2DM bone defects enhances angiogenesis at both early and late stages of regeneration and osteogenesis as well as osteoclastogenesis at late stages. The mechanisms how FGF-9 regulates angiogenesis in bone repair are largely unknown. However, it has been demonstrated that application of FGF-9 induces VEGF expression suggesting a linkage between FGF-9 signaling and VEGF expression [64]. Interestingly, FGF-9 has been shown to induce angiogenesis without directly targeting endothelial cells. In an ischemia hind limb model FGF-9 was found to target non-endothelial mesenchymal cell populations [67] indicating that FGF-9 could also influence mesenchymal populations in the bone which could further lead to enhanced angiogenesis accompanied by increased osteoid formation. Furthermore, enhanced angiogenesis could be accompanied with osteoclasts invasion resulting in increased bone remodeling.

VEGFA and FGF-9 are both known to facilitate osteogenesis and angiogenesis during skeletal development acting in various ways. In our study, FGF-9 application was more potent to enhance angiogenesis and bone regeneration than VEGFA. It has been shown that VEGFA is upregulated in FGF-9 enriched environment [28,68]. Moreover, Hung et. al. have demonstrated that FGF-9 is sufficient to induce *Vegf* expression during skeletal development [64]. This leads to the conclusion that FGF-9 co-stimulates a VEGFA expression and therefore may surpass the osteogenic effect of VEGFA application. Future experiments will further optimize applied concentrations of both growth factors and may prove to be a valuable tool to foster bone regeneration in compromised clinical situations such as type 2 diabetic condition.

Kawaguchi et al. have carried out a first clinical study utilizing local application of FGF-2 to accelerate bone regeneration in human tibial shaft fractures [69]. They could demonstrate a significant improvement of bone regeneration in FGF-2 treated patients, which raises hopes that the fibroblast growth factor family member FGF-9 might also be successfully utilized in a similar clinical setting.

## Conclusions

In the current study, we demonstrated that angiogenesis, osteogenesis and bone remodelling are impaired in T2DM bone regeneration. In T2DM bony defects, important proteins involved in proliferation, osteoblast differentiation and angiogenesis are altered. Osteogenesis and angiogenesis could be enhanced by local application of VEGFA into the bony defect and reconstituted by application of FGF-9 to levels of WT mice. Therefore, we expect a positive response in large animal experiments to expedite a clinical implementation of FGF-9. We imagine a clinical situation where local FGF-9 could be administered to otherwise challenged bony defects in diabetic patients. Considering the epidemiological impact of diabetic associated bony defects, local FGF-9 treatment based on this strategy would not only clinically improve the current unsatisfying treatment situation, but also reduce the huge economic burden resulting from disabilities and expensive cascades of unsuccessful treatment approaches.

## Supporting Information

S1 Fig Unicortical defects in *db-/db-* and WT mice heal by intramembranous ossification. (A) Safranin-O staining of tibial proximal epiphyseal plate showing cartilage (red) and bone (blue) in WT animal as control. (B) Safranin-O staining of a tibial defect 7dpO in *db* ^-^/*db* ^-^ mice reveals no cartilage in the defect area similar to WT mice 7dpO (C). Yellow dashed line indicates collagen sponge.
(DOCX)

## References

[1] Gaston MS, Simpson AHRW (2007) Inhibition of fracture healing. J Bone Joint Surg Br 89: 1553–1560. 18057352 10.1302/0301-620X.89B12.19671

[2] Retzepi M, Donos N (2010) The effect of diabetes mellitus on osseous healing. Clin Oral Implants Res 21: 673–681. 10.1111/j.1600-0501.2010.01923.x 20465554

[3] Daneman D (2006) Type 1 diabetes. Lancet 367: 847–858. 16530579 10.1016/S0140-6736(06)68341-4

[4] Isidro ML, Ruano B (2010) Bone disease in diabetes. Curr Diabetes Rev 6: 144–155. 20380629 10.2174/157339910791162970

[5] Khazai NB, Beck GR, Umpierrez GE (2009) Diabetes and fractures: an overshadowed association. Curr Opin Endocrinol Diabetes Obes 16: 435–445. 10.1097/MED.0b013e328331c7eb 19779334 PMC3746497

[6] Cozen L (1972) Does diabetes delay fracture healing? Clin Orthop Relat Res 82: 134–140. 5011018

[7] Loder RT (1988) The influence of diabetes mellitus on the healing of closed fractures. Clin Orthop Relat Res: 210–216. 3289812

[8] Perlman MH, Thordarson DB (1999) Ankle fusion in a high risk population: an assessment of nonunion risk factors. Foot ankle Int / Am Orthop Foot Ankle Soc [and] Swiss Foot Ankle Soc 20: 491–496. 10.1177/107110079902000805 10473059

[9] Beam HA, Parsons JR, Lin SS (2002) The effects of blood glucose control upon fracture healing in the BB Wistar rat with diabetes mellitus. J Orthop Res 20: 1210–1216. 12472231 10.1016/S0736-0266(02)00066-9

[10] Gebauer GP, Lin SS, Beam HA, Vieira P, Parsons JR (2002) Low-intensity pulsed ultrasound increases the fracture callus strength in diabetic BB Wistar rats but does not affect cellular proliferation. J Orthop Res 20: 587–592. 12038635 10.1016/S0736-0266(01)00136-X

[11] Tyndall WA, Beam HA, Zarro C, O’Connor JP, Lin SS (2003) Decreased platelet derived growth factor expression during fracture healing in diabetic animals. Clin Orthop Relat Res: 319–330. 10.1097/00003086-200303000-00043 12616077

[12] Gandhi A, Doumas C, Dumas C, O’Connor JP, Parsons JR, et al (2006) The effects of local platelet rich plasma delivery on diabetic fracture healing. Bone 38: 540–546. 16368279 10.1016/j.bone.2005.10.019

[13] Weyer C, Funahashi T, Tanaka S, Hotta K, Matsuzawa Y, et al. (2013) Hypoadiponectinemia in Obesity and Type 2 Diabetes: Close Association with Insulin Resistance and Hyperinsulinemia. 10.1210/jcem.86.5.7463 11344187

[14] He H, Liu R, Desta T, Leone C, Gerstenfeld LC, et al (2004) Diabetes causes decreased osteoclastogenesis, reduced bone formation, and enhanced apoptosis of osteoblastic cells in bacteria stimulated bone loss. Endocrinology 145: 447–452. 14525917 10.1210/en.2003-1239

[15] Wang F, Song Y, Li D, Li C, Wang Y, et al (2010) Type 2 diabetes mellitus impairs bone healing of dental implants in GK rats. Diabetes Res Clin Pract 88: e7–9. 10.1016/j.diabres.2010.01.017 20138383

[16] Kawaguchi H, Kurokawa T, Hanada K, Hiyama Y, Tamura M, et al (1994) Stimulation of fracture repair by recombinant human basic fibroblast growth factor in normal and streptozotocin-diabetic rats. Endocrinology 135: 774–781. 8033826 10.1210/endo.135.2.8033826

[17] Al-Zube L, Breitbart EA, O’Connor JP, Parsons JR, Bradica G, et al (2009) Recombinant human platelet-derived growth factor BB (rhPDGF-BB) and beta-tricalcium phosphate/collagen matrix enhance fracture healing in a diabetic rat model. J Orthop Res 27: 1074–1081. 10.1002/jor.20842 19170096

[18] Franceschi RT (2005) Biological Approaches to Bone Regeneration by Gene Therapy. J Dent Res 84: 1093–1103. 16304438 10.1177/154405910508401204

[19] Gerber HP, Vu TH, Ryan AM, Kowalski J, Werb Z, et al (1999) VEGF couples hypertrophic cartilage remodeling, ossification and angiogenesis during endochondral bone formation. Nat Med 5: 623–628. 10371499 10.1038/9467

[20] Rowe NM, Mehrara BJ, Luchs JS, Dudziak ME, Steinbrech DS, et al (1999) Angiogenesis during mandibular distraction osteogenesis. Ann Plast Surg 42: 470–475. 10340853 10.1097/00000637-199905000-00002

[21] Vu TH, Shipley JM, Bergers G, Berger JE, Helms JA, et al (1998) MMP-9/gelatinase B is a key regulator of growth plate angiogenesis and apoptosis of hypertrophic chondrocytes. Cell 93: 411–422. 9590175 10.1016/s0092-8674(00)81169-1 PMC2839071

[22] Glowacki J (1998) Angiogenesis in fracture repair. Clin Orthop Relat Res: S82–9. 10.1097/00003086-199810001-00010 9917629

[23] Santana RB, Xu L, Chase HB, Amar S, Graves DT, et al (2003) A role for advanced glycation end products in diminished bone healing in type 1 diabetes. Diabetes 52: 1502–1510. 12765963 10.2337/diabetes.52.6.1502

[24] Amin AH, Abd Elmageed ZY, Nair D, Partyka MI, Kadowitz PJ, et al (2010) Modified multipotent stromal cells with epidermal growth factor restore vasculogenesis and blood flow in ischemic hind-limb of type II diabetic mice. Lab Invest 90: 985–996. 10.1038/labinvest.2010.86 20440273 PMC3154725

[25] Galiano RD, Tepper OM, Pelo CR, Bhatt KA, Callaghan M, et al (2004) Topical vascular endothelial growth factor accelerates diabetic wound healing through increased angiogenesis and by mobilizing and recruiting bone marrow-derived cells. Am J Pathol 164: 1935–1947. 15161630 10.1016/S0002-9440(10)63754-6 PMC1615774

[26] Street J, Bao M, deGuzman L, Bunting S, Peale F V, et al (2002) Vascular endothelial growth factor stimulates bone repair by promoting angiogenesis and bone turnover. Proc Natl Acad Sci U S A 99: 9656–9661. 12118119 10.1073/pnas.152324099 PMC124965

[27] Behr B, Longaker MT, Quarto N (2010) Differential activation of canonical Wnt signaling determines cranial sutures fate: a novel mechanism for sagittal suture craniosynostosis. Dev Biol 344: 922–940. 10.1016/j.ydbio.2010.06.009 20547147

[28] Behr B, Leucht P, Longaker MT, Quarto N (2010) Fgf-9 is required for angiogenesis and osteogenesis in long bone repair. Proc Natl Acad Sci U S A 107: 11853–11858. 10.1073/pnas.1003317107 20547837 PMC2900703

[29] Fakhry A, Ratisoontorn C, Vedhachalam C, Salhab I, Koyama E, et al (2005) Effects of FGF-2/-9 in calvarial bone cell cultures: differentiation stage-dependent mitogenic effect, inverse regulation of BMP-2 and noggin, and enhancement of osteogenic potential. Bone 36: 254–266. 15780951 10.1016/j.bone.2004.10.003

[30] Behr B, Tang C, Germann G, Longaker MT, Quarto N (2011) Locally applied vascular endothelial growth factor A increases the osteogenic healing capacity of human adipose-derived stem cells by promoting osteogenic and endothelial differentiation. Stem Cells 29: 286–296. 10.1002/stem.581 21732486 PMC3400547

[31] Horvat S, Bünger L (1999) Polymerase chain reaction-restriction fragment length polymorphism (PCR-RFLP) assay for the mouse leptin receptor (Leprdb) mutation. Lab Anim 33: 380–384. 10778787 10.1258/002367799780487850

[32] Behr B, Sorkin M, Manu A, Lehnhardt M, Longaker MT, et al (2011) Fgf-18 is required for osteogenesis but not angiogenesis during long bone repair. Tissue Eng Part A 17: 2061–2069. 10.1089/ten.TEA.2010.0719 21457097 PMC3142654

[33] Ralis H, Ralis Z (1975) A simple method for demonstration of osteoid in paraffin sections. Med Lab Technol 32: 203–213. 51466

[34] Kahveci Z, Minbay FZ, Cavusoglu L (2000) Safranin O staining using a microwave oven. Biotech Histochem 75: 264–268. 11131567 10.3109/10520290009085130

[35] Iwaki A, Jingushi S, Oda Y, Izumi T, Shida JI, et al (1997) Localization and quantification of proliferating cells during rat fracture repair: detection of proliferating cell nuclear antigen by immunohistochemistry. J Bone Miner Res 12: 96–102. 9240731 10.1359/jbmr.1997.12.1.96

[36] Pavlidis D, Bourauel C, Rahimi A, Götz W, Jäger A (2009) Proliferation and differentiation of periodontal ligament cells following short-term tooth movement in the rat using different regimens of loading. Eur J Orthod 31: 565–571. 10.1093/ejo/cjp053 19635744

[37] Ding D, Li L, Song Y, DU G, Wei X, et al (2013) MAPK-ERK1/2 signaling pathway regulates osteogenic gene expression in rat osteoblasts in vitro. Nan fang yi ke da xue xue bao J South Med Univ 33: 1432–1436. 24144741

[38] Stepanovic V, Awad O, Jiao C, Dunnwald M, Schatteman GC (2003) Leprdb diabetic mouse bone marrow cells inhibit skin wound vascularization but promote wound healing. Circ Res 92: 1247–1253. 12730094 10.1161/01.RES.0000074906.98021.55

[39] Michaels J, Churgin SS, Blechman KM, Greives MR, Aarabi S, et al (2007) db/db mice exhibit severe wound-healing impairments compared with other murine diabetic strains in a silicone-splinted excisional wound model. Wound Repair Regen 15: 665–670. 17971012 10.1111/j.1524-475X.2007.00273.x

[40] Lu H, Kraut D, Gerstenfeld LC, Graves DT (2003) Diabetes interferes with the bone formation by affecting the expression of transcription factors that regulate osteoblast differentiation. Endocrinology 144: 346–352. 12488363 10.1210/en.2002-220072

[41] Williams GA, Callon KE, Watson M, Costa JL, Ding Y, et al (2011) Skeletal phenotype of the leptin receptor-deficient db/db mouse. J Bone Miner Res 26: 1698–1709. 10.1002/jbmr.367 21328476

[42] Hickman J, McElduff A (1989) Insulin promotes growth of the cultured rat osteosarcoma cell line UMR-106–01: an osteoblast-like cell. Endocrinology 124: 701–706. 2536316 10.1210/endo-124-2-701

[43] Guarneri MP, Weber G, Gallia P, Chiumello G (1993) Effect of insulin treatment on osteocalcin levels in diabetic children and adolescents. J Endocrinol Invest 16: 505–509. 8227979 10.1007/BF03348892

[44] Ferron M, Wei J, Yoshizawa T, Del Fattore A, DePinho RA, et al (2010) Insulin signaling in osteoblasts integrates bone remodeling and energy metabolism. Cell 142: 296–308. 10.1016/j.cell.2010.06.003 20655470 PMC2910411

[45] Botolin S, McCabe LR (2006) Chronic hyperglycemia modulates osteoblast gene expression through osmotic and non-osmotic pathways. J Cell Biochem 99: 411–424. 16619259 10.1002/jcb.20842

[46] Wang A, Midura RJ, Vasanji A, Wang AJ, Hascall VC (2014) Hyperglycemia diverts dividing osteoblastic precursor cells to an adipogenic pathway and induces synthesis of a hyaluronan matrix that is adhesive for monocytes. J Biol Chem 289: 11410–11420. 10.1074/jbc.M113.541458 24569987 PMC4036277

[47] Tamura Y, Kawao N, Okada K, Yano M, Okumoto K, et al (2013) Plasminogen activator inhibitor-1 is involved in streptozotocin-induced bone loss in female mice. Diabetes 62: 3170–3179. 10.2337/db12-1552 23715621 PMC3749344

[48] Catalfamo DL, Britten TM, Storch DL, Calderon NL, Sorenson HL, et al (2013) Hyperglycemia induced and intrinsic alterations in type 2 diabetes-derived osteoclast function. Oral Dis 19: 303–312. 10.1111/odi.12002 24079914 PMC3800028

[49] Cunha JS, Ferreira VM, Maquigussa E, Naves MA, Boim MA (2014) Effects of high glucose and high insulin concentrations on osteoblast function in vitro. Cell Tissue Res. 10.1007/s00441-014-1913-x 24859221

[50] Xu F, Ye Y, Dong Y, Guo F, Chen A, et al (2013) Inhibitory effects of high glucose/insulin environment on osteoclast formation and resorption in vitro. J Huazhong Univ Sci Technolog Med Sci 33: 244–249. 10.1007/s11596-013-1105-z 23592138

[51] Fadini GP, Miorin M, Facco M, Bonamico S, Baesso I, et al (2005) Circulating endothelial progenitor cells are reduced in peripheral vascular complications of type 2 diabetes mellitus. J Am Coll Cardiol 45: 1449–1457. 15862417 10.1016/j.jacc.2004.11.067

[52] Loomans CJM, de Koning EJP, Staal FJT, Rookmaaker MB, Verseyden C, et al (2004) Endothelial progenitor cell dysfunction: a novel concept in the pathogenesis of vascular complications of type 1 diabetes. Diabetes 53: 195–199. 14693715 10.2337/diabetes.53.1.195

[53] D’Souza DR, Salib MM, Bennett J, Mochin-Peters M, Asrani K, et al (2009) Hyperglycemia regulates RUNX2 activation and cellular wound healing through the aldose reductase polyol pathway. J Biol Chem 284: 17947–17955. 10.1074/jbc.M109.002378 19383984 PMC2709386

[54] Keyszer G, Lambiri I, Nagel R, Keysser C, Keysser M, et al (1999) Circulating levels of matrix metalloproteinases MMP-3 and MMP-1, tissue inhibitor of metalloproteinases 1 (TIMP-1), and MMP-1/TIMP-1 complex in rheumatic disease. Correlation with clinical activity of rheumatoid arthritis versus other surrogate markers. J Rheumatol 26: 251–258. 9972954

[55] Chen Q, Jin M, Yang F, Zhu J, Xiao Q, et al (2013) Matrix metalloproteinases: inflammatory regulators of cell behaviors in vascular formation and remodeling. Mediators Inflamm 2013: 928315 10.1155/2013/928315 23840100 PMC3694547

[56] Scheede-Bergdahl C, Bergdahl A, Schjerling P, Qvortrup K, Koskinen SO, et al (2014) Exercise-induced regulation of matrix metalloproteinases in the skeletal muscle of subjects with type 2 diabetes. Diab Vasc Dis Res 11: 324–334. 10.1177/1479164114535943 24903024

[57] Han SY, Jee YH, Han KH, Kang YS, Kim HK, et al (2006) An imbalance between matrix metalloproteinase-2 and tissue inhibitor of matrix metalloproteinase-2 contributes to the development of early diabetic nephropathy. Nephrol Dial Transplant 21: 2406–2416. 16728425 10.1093/ndt/gfl238

[58] Huber J, Kiefer FW, Zeyda M, Ludvik B, Silberhumer GR, et al (2008) CC chemokine and CC chemokine receptor profiles in visceral and subcutaneous adipose tissue are altered in human obesity. J Clin Endocrinol Metab 93: 3215–3221. 10.1210/jc.2007-2630 18492752

[59] Yamamoto T, Eckes B, Mauch C, Hartmann K, Krieg T (2000) Monocyte chemoattractant protein-1 enhances gene expression and synthesis of matrix metalloproteinase-1 in human fibroblasts by an autocrine IL-1 alpha loop. J Immunol 164: 6174–6179. 10843667 10.4049/jimmunol.164.12.6174

[60] Tatsuyama K, Maezawa Y, Baba H, Imamura Y, Fukuda M (2000) Expression of various growth factors for cell proliferation and cytodifferentiation during fracture repair of bone. Eur J Histochem 44: 269–278. 11095098

[61] Zelzer E, Olsen BR (2005) Multiple roles of vascular endothelial growth factor (VEGF) in skeletal development, growth, and repair. Curr Top Dev Biol 65: 169–187. 15642383 10.1016/S0070-2153(04)65006-X

[62] Maes C, Stockmans I, Moermans K, Van Looveren R, Smets N, et al (2004) Soluble VEGF isoforms are essential for establishing epiphyseal vascularization and regulating chondrocyte development and survival. J Clin Invest 113: 188–199. 14722611 10.1172/JCI19383 PMC312596

[63] Yang Y-Q, Tan Y-Y, Wong R, Wenden A, Zhang L-K, et al (2012) The role of vascular endothelial growth factor in ossification. Int J Oral Sci 4: 64–68. 22722639 10.1038/ijos.2012.33 PMC3412670

[64] Hung IH, Yu K, Lavine KJ, Ornitz DM (2007) FGF9 regulates early hypertrophic chondrocyte differentiation and skeletal vascularization in the developing stylopod. Dev Biol 307: 300–313. 17544391 10.1016/j.ydbio.2007.04.048 PMC2267922

[65] Garofalo S, Kliger-Spatz M, Cooke JL, Wolstin O, Lunstrum GP, et al (1999) Skeletal dysplasia and defective chondrocyte differentiation by targeted overexpression of fibroblast growth factor 9 in transgenic mice. J Bone Miner Res 14: 1909–1915. 10571691 10.1359/jbmr.1999.14.11.1909

[66] Harada M, Murakami H, Okawa A, Okimoto N, Hiraoka S, et al (2009) FGF9 monomer-dimer equilibrium regulates extracellular matrix affinity and tissue diffusion. Nat Genet 41: 289–298. 10.1038/ng.316 19219044 PMC2676118

[67] Frontini MJ, Nong Z, Gros R, Drangova M, O’Neil C, et al (2011) Fibroblast growth factor 9 delivery during angiogenesis produces durable, vasoresponsive microvessels wrapped by smooth muscle cells. Nat Biotechnol 29: 421–427. 10.1038/nbt.1845 21499246

[68] Teishima J, Yano S, Shoji K, Hayashi T, Goto K, et al (2014) Accumulation of FGF9 in prostate cancer correlates with epithelial-to-mesenchymal transition and induction of VEGF-A expression. Anticancer Res 34: 695–700. 24511001

[69] Kawaguchi H, Oka H, Jingushi S, Izumi T, Fukunaga M, et al (2010) A local application of recombinant human fibroblast growth factor 2 for tibial shaft fractures: A randomized, placebo-controlled trial. J Bone Miner Res 25: 2735–2743. 10.1002/jbmr.146 20533373

