# Supplementary material for: Application of VEGFA and FGF-9 Enhances Angiogenesis, Osteogenesis and Bone Remodeling in Type 2 Diabetic Long Bone Regeneration
Source: PLoS One. 2015 Mar 5;10(3):e0118823. doi: 10.1371/journal.pone.0118823 (PMC4350939; doi:10.1371/journal.pone.0118823)
Supplement: S1 Fig — (A) Safranin-O staining of tibial proximal epiphyseal plate showing cartilage (red) and bone (blue) in WT animal as control. (B) Safranin-O staining of a tibial defect 7dpO in db -/db - mice reveals no cartilage in the defect area similar to WT mice 7dpO (C). Yellow dashed line indicates collagen sponge. (DOCX) [file pone.0118823.s001.docx]

**
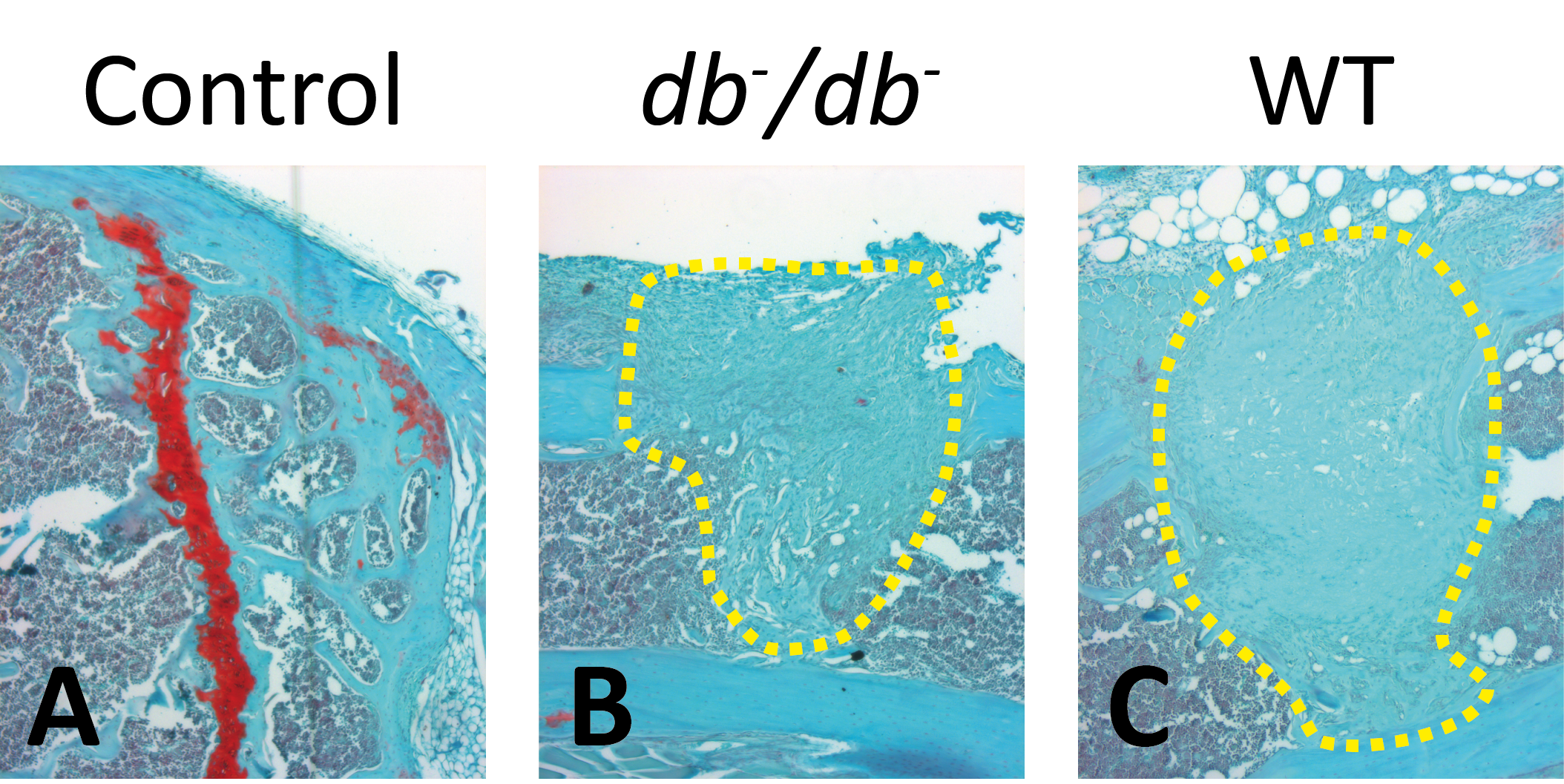
**

**Fig. S1: Unicortical defects in *db^-^/db^-^* and WT mice heal by intramembranous ossification.** (A) Safranin-O staining of tibial proximal epiphyseal plate showing cartilage (red) and bone (blue) in WT animal as control. (B) Safranin-O staining of a tibial defect 7dpO in *db^-^/db^-^* mice reveals no cartilage in the defect area similar to WT mice 7dpO (C). Yellow dashed line indicates collagen sponge.
